# Optimisation of systems, safety and efficiency using simulation: a qualitative exploration of the value proposition for healthcare leaders

**DOI:** 10.1186/s41077-025-00378-8

**Published:** 2025-10-23

**Authors:** Sharon Clipperton, Leah McIntosh, Sarah Janssens

**Affiliations:** 1 Mater Health, Mater Misericordiae, Brisbane, Australia; 2 Mater Misericordiae Brisbane Ltd, Mothers Babies and Womens’ Health Services, Brisbane, Australia; 3 Faculty of Medicine, University of Queensland, Brisbane, Australia

**Keywords:** Simulation-based testing, Quality improvement, Leaders, Value, Healthcare

## Abstract

**Background:**

Simulation-based testing (SBT) is increasingly recognised as a strategic tool for enhancing healthcare safety, efficiency, and system readiness. Despite its growing use, the perspectives of healthcare leaders—key stakeholders in sustaining simulation services—remain underexplored.

This study aimed to understand how healthcare leaders perceive the value of SBT, identify drivers for engagement, and explore how simulation activities can be aligned with leadership priorities to support quality improvement.

**Methods:**

A qualitative, phenomenological approach was used, involving semi-structured interviews with nine healthcare leaders from executive, clinical, and infrastructure roles within a large Australian health service. Thematic analysis was conducted to identify key themes from the interview data.

**Results:**

Three major themes emerged:

Optimising operations—Leaders valued SBT for its ability to test real-world workflows, uncover latent safety threats, and ensure regulatory and licensing readiness. SBT was seen as a diagnostic tool that supports operational assurance and futureproofing.

Collaborative design—Leaders emphasised the importance of engaging end users in simulation activities to ensure relevance, foster shared understanding, and support implementation. SBT was also valued for its role in experiential orientation and team cohesion.

Reporting and recommendations—Timely, structured, and risk-stratified reporting was critical for decision-making. Leaders preferred concise executive summaries and actionable recommendations aligned with project goals and resource constraints.

**Conclusion:**

Healthcare leaders demonstrated a sophisticated understanding of SBT’s strategic applications beyond education. They recognised its value in improving routine operations, supporting compliance, and informing high-stakes decisions. Simulation faculty can enhance the impact and sustainability of SBT by aligning activities with leadership priorities, ensuring timely communication, and delivering focused, actionable insights. These findings offer guidance for embedding SBT into broader quality improvement and organisational strategies. Future research should explore the generalisability of these findings across less mature simulation services and further investigate SBT’s role in regulatory compliance.

## Introduction

Simulation service sustainability relies on the perceived value by healthcare organisation leaders. Despite this, leadership perspectives are rarely explored by simulation researchers. This research describes the value of simulation-based testing (SBT) from the leader’s perspective and identifies key strategies to enhance leader support for SBT. For simulation faculty, this research describes how to align activities with leadership priorities and foster the targeted application of simulation techniques to improve safety, efficiency and experiences.

## Background

**Table:** 

What we know: • Simulation is a tool that supports quality improvement • Simulation services need to demonstrate value to healthcare leadership to ensure sustainability • Leaders navigate complex healthcare landscapes that demand continuous improvement • Leaders are uniquely positioned to support integration of simulation and quality improvement	

Since its introduction, simulation and simulation programs have needed to demonstrate value, prove return on investment, and create sustainable models of service delivery that appeal to funders, hospital leaders and administrators. Whilst this remains challenging for simulation programs, it has prompted the evolution of simulation, recognising the potential for simulation to add value through the exploration of systems/processes, safety and performance of organisations in addition to more traditional or expected applications such as staff development and education.

Simulation enthusiasts have long been calling for widespread recognition of the powerful contribution simulation can make to patient safety and quality improvement [1, 2]. The growing literature indicates that educational simulation activities undeniably contribute to quality improvement through the identification of latent safety threats, system and process inefficiencies and unsatisfactory user experiences [3–5]. Despite this, SBT has been an underutilised and undercapitalised resource [6, 7].Weldon et al. suggest the educational roots of simulation have led to a failure to recognise key differences, resulting in misinterpretation of SBT activity purpose and intention. This has delayed the development of robust frameworks and guidance, which has stunted meaningful integration with quality and safety systems [7].

Recognising and reconciling the interplay between quality improvement and education outcomes, the emerging literature has focused on developing the appropriate vocabulary, frameworks and guidance to clarify these concepts. Brazil et al. describe ‘translational simulation’; highlighting that education and quality improvement are not mutually exclusive, asserting that system and process improvement and development of individuals, teams and relationships explored during simulation can translate to improved patient outcomes. In attempting to provide common understanding, Weldon et al. add to this, coining the term ‘transformative simulation’, identifying that simulation is a tool that creates shared ‘understanding, insight and learning’, transforming healthcare through both pedagogical (education) and non-pedagogical (systems and process improvement) simulation. This research paper is focused on simulation activities conducted for non-pedagogical purposes. Various terms describe non-pedagogical simulation such as ‘patient safety and system integration’ (PSSI) [8] simulation and ‘simulation based clinical system testing’ (SbCST) [9–11]. For the purposes of this paper, we have chosen the term ‘simulation based testing’ to capture and describe non-pedagogical activities that include both simulation for diagnosing system and process weaknesses and simulation for creating and testing interventions and the integration of fit for purpose solutions. Regardless of the terminology used, the literature which positions the synergy between simulation and quality improvement describes a number of examples and case studies of simulation activities that recognise both tangible and intangible benefits and outcomes [11, 12]. These projects have focused on informing facility design, testing new and established systems/processes and the evaluation of operational preparedness with benefits realised including cost avoidance, cost reduction, improved efficiency and improved resource utilisation [9, 12–16].

This idea, that simulation is a tool that can be deployed as part of a strategy to support healthcare organisations to improve safety, increase efficiency, reduce wastage and improve satisfaction, is appealing for healthcare leaders [7, 17]. Healthcare leaders navigate complex landscapes involving infrastructure, finance, regulatory/governing bodies, human resources and patient outcomes with demands to reduce costs and ensure high-value care is delivered in a continuous cycle of striving for improvement [17]. In addition, healthcare leaders play a pivotal role in determining the vision for healthcare organisations, influencing cultures that promote innovation, collaboration and positive outcomes balanced with resource allocation and prioritisation of strategies [18]. In this sense, hospital leaders are in a unique position to operationalise the integration of simulation and quality improvement, harnessing SBT and driving system-wide improvement [19].

Whilst the faculty experience of implementing simulation programs has been described [20, 21] a gap in the understanding of the hospital leader’s perspective of the value proposition of SBT remains. This study explores healthcare leader perceptions of SBT, examining drivers for engagement and interpretations of outcomes to inform the design of high-impact activities by simulation faculty. Within this article, we use the word ‘leaders’ to describe individuals in healthcare leadership positions from executive, governance, clinical, and built environment backgrounds who have previously engaged the simulation service for SBT.

## Aim

This research aims to understand the value proposition of SBT for healthcare leaders by exploring the drivers for engagement and their perceptions of the application of SBT and project outcomes.

## Methods

To ensure transparent reporting of the research design, analysis and reporting, the COREQ guidelines and checklist [22] were applied.

### Study design

This study employed a phenomenological approach using semi-structured interviews to explore healthcare leaders’ perceptions of SBT. A social constructivist lens informed the study design, recognising the rich experiences and interactions of participants and researchers shape knowledge creation. The research team has extensive experience with simulation-based education and SBT; as such, reflexivity was maintained, with the researchers acknowledging their dual roles as simulation faculty and researchers could introduce bias.

### Researcher positioning

The authors all have educational, clinical and content expertise with over a decade of experience in this area. SC is an RN/RM, and LMC is an RN, employed as education coordinators. SJ is a staff specialist, experienced educator and clinical administrator, all working in a large metropolitan tertiary facility. The researchers acknowledge that their experience across both educational and systems-focused simulation has shaped their perspective on the value of simulation. This positioning enabled deep analysis of the research data while requiring critical reflection to minimise bias and ensure credibility of findings.

### Ethical considerations

Project approved by Mater Misericordiae Ltd Human Research Ethics Committee HREC/MML/81095 (V3).

### Recruitment

Purposive sampling was used to identify leaders across the organisation who had previously engaged the SBT service (Table 1). These leaders provided representation from facility leadership, clinical leadership, capital works, and hospital quality and safety. All were executive leaders, directors and/or manager leaders within a statewide healthcare organisation and were employed at different sites/facilities across the state (Queensland, Australia).

**Table 1:** Participant demographics

Identifier	Role and department	Simulation-based testing activity
P1	Clinical Coordinator Quality & Safety	Workflow design
P2	Clinical Lead Clinical Services (Allied Health)	Workflow design, operational readiness
P3	Director Clinical Services	Procedure rehearsal, workflow design, incident investigation
P4	Director Capital Works	Prototype design, facility and systems testing, operational readiness
P5	Director Clinical Services	Incident investigation, systems testing operational readiness, procedure design,
P6	Assistant Director Clinical Services	Facility testing
P7	Director Clinical Services	Procedure design, systems testing operational readiness
P8	Director Quality & Safety	Procedure design
P9	Director Built Environment	Prototype testing, facility testing and workflow design

Invitations were sent to 15 leaders via email, with 8 interviews agreed to. Snowball recruitment resulted in one additional participant, making a total of 9 interviews. All participants provided written informed consent. Participation was voluntary, and non-reply to the email invitation was assumed as declining to participate.

### Interviews

In preparation, the outcome report for the SBT activity the leader requested/sponsored was provided to the interviewee approximately 7 days prior to the interview.

An interview guide was developed and piloted with P1. The pilot refined the wording of questions to improve relevance and question interpretation [23]. Pilot data was included in the study. Questions gathered leaders’ perspectives on the SBT service, focusing on key areas such as purpose and rationale for the activity, overarching vision, delivery of SBT activity, how recommendations were reported, and the overall impact on the project. The interview guide is provided in Appendix 1.

One on one interviews were conducted within business hours by a single interviewer (SC) and recorded via Microsoft Teams. Interviews were approximately 30 min in duration, providing almost 5 h of data subsequently transcribed by researchers LM and SC. Data saturation became apparent during the final interviews, and the researchers identified that further leadership recruitment was not required. Transcribed interviews were reviewed by the interviewer (SC) to confirm data accuracy. Recordings and transcriptions were securely stored in a password protected database.

#### Analysis

The transcripts were analysed by all 3 researchers (SC, LM and SJ), using inductive thematic analysis with reflexivity following the six steps: (1) familiarisation, (2) coding, (3) generation of initial themes, (4) refining and defining (5) naming themes, and (6) writing the report [24].

## Results

Nine semi-structured interviews were conducted. All participants hold senior-level positions as described in Table 1.

The following themes were identified through reflexive thematic analysis of the data.

1. Optimising operations: Captures how leaders identified benefits of SBT to streamline their healthcare systems. Including what they wanted to test, who they wanted to test with, and what findings they found useful.
2. Collaborative design: Describes leaders’ appreciation for the benefits gained from exploring multiple professional viewpoints and implementation benefits gained by engaging end users prior to process finalisation.
3. Reporting outcomes and recommendations: Identifies what leaders value about or in reports, including timing, content, and risk stratification preferences.

The following sections expand upon each of these themes in turn, and further examples of relevant quotes can be found in Table 2.

**Table 2:** Themes and relevant quotes

Optimise operations	Leaders identified benefits of SBT to streamline their healthcare systems. Including what they wanted to test, who they wanted to test with, and what findings they found useful P3 “simulation should be used to get the everyday things right, the routine workflow, not just saving it for the fun and exciting new buildings” P4 “…areas that require testing…. the design plan for any areas that could be challenging, systems testing for staff…codes… and standard workflow such as movement around the bedside and equipment in and around rooms” P7 “to enable us to continue to get a licence that didn’t inhibit the services we’re already doing, ….to protect the business from being limited” P5 “Actually stepping it out with a group of people helped to visualise what the roadblocks were” P1 “it has to be performed under conditions that are as close as possible to the real world otherwise people don’t perform as they usually would” P8 “I think it gives you a view through a different lens… the opportunity to think about things differently” P9 “It gives those people that are approving the go live a sense of confidence… making decisions that a third party has gone through and has done the checks” P9 “It’s not just the business that’s saying it. It’s someone independent that’s going, OK, we can walk in and make this happen… someone else needs to be able to come in and use the space and the report just gives the executive some confidence that the third party, even though it’s an internal party, comes in” P2 “This was a high stakes activity and failure was not an option …we needed to understand the risks … and potential disruptions”
Collaborative design	Leaders’ appreciation for the benefits gained from exploring multiple professional viewpoints and implementation benefits gained by engaging end users prior to process finalisation P9 “having end users there is super helpful because they know that they understand their own work as they would do it. Whereas we, we might have a vision of how things might look but not actually function” P1 “we need new and fresh eyes from the people engaging in the tasks and asking the questions that we haven’t thought of” P2 “we’ve tested this, we know what to do and we know we can do it’. Things can get flustered, but ‘no its ok, we’ve got our process, we’ve got it set, we’re good’” P5 “Well, we, in theory think it should work, that’s why! But in reality, its different, …at 10:00 in the morning, that’s a completely different story” P4 “…also from the perspective of staff seeing what will and won’t work for them so they can refine their own processes. It also helps us too, to explain, often when staff go in, they’re like ‘well that’s dumb why did they do it like that?’ and it gives us an opportunity to say ‘okay, this is why… it has been designed this way because…’” P8 “It just brings everyone together and helps everyone understand why it is like it is and… whether changes are viable…”
Reporting outcomes and recommendations	What leaders value about reports, including timing, content, and risk stratification preferences P4 “That was definitely part of the go no go. I think it will be a fundamental part of the go no go process, moving forwards, we are asked ‘have you done your simulations?’ ‘What simulations have you done?’ and ‘the feedback from the simulation team, what is that?’, ‘are they comfortable?” P1 “… the [activity] report took a while to come back. We need to be able to access the team and have a quick turnaround on the report, sometimes in the [hospital] we need to step projects up pretty quickly” P5 “I actually got more out of the informal huddle at the end then sometimes as a report because the narrative helped me understand a little bit more than if I had read it on a pure action plan” P4 “the project team seems to really like… is that walking away at the end of that day with pretty much having a very good idea of what those recommendations are, so that if the report is 48 h or 72 h, they’re pretty much starting on some of them recommendations right then” P7 “So looking at really… what is a patient or staff safety issue, what is nice to have rather than everything” P3 “what is the most impactful is the first executive summary, these are some of the things that we would recommend changing or improving right now” P4 “A barrier is not having the space ready, systems not functional makes it very difficult to trust the outcomes” P9 “It gives those people that are approving the go-live a sense of confidence… making decisions that a third party has gone through and done the checks”

### Theme 1: Optimising operations

Participants identified the optimisation of operations and operational readiness as a key driver of engagement with SBT. Leaders described the key features that promote this optimisation as physical simulation of work as done, independent assessment/assurance, understanding and application of licensing and regulatory requirements, and the ability to replicate and uncover latent safety threats within the system.

Leaders described the need for simulations reflecting usual workflows, reassurance that systems and processes work as intended during emergent situations, equipment availability and suitability, and an assessment of operation preparation/readiness. Interestingly, leaders emphasised the need for simulation activities that examine routine ‘work as done’ ensuring end users are provided with opportunities to improve everyday operations.

P3- Simulation should be used to get the everyday things right, the routine workflow, not just saving it for the fun and exciting new buildings

Interestingly, leaders discussed accessing SBT activities to ensure organisational preparedness related to licensing, regulations and credentialling. SBT provides opportunities to test systems, processes and environments through targeted scenarios, ensuring compliance and mitigating the risk of withdrawal of licensing/accreditation, which would limit service delivery. Leaders recognise the value of SBT in supplementing the reports, evidence, and assurance requirements related to relevant application and compliance requirements.

P7- to enable us to continue to get a license that didn’t inhibit the services we’re already doing….. to protect the business from being limited

In addition to workflows, leaders also described the utility of SBT simulation to understand service scope, capability and scalability. The ability for simulation to replicate situations that place systems under stress informs decisions about future proofing and service growth.

Strategies that increased leader confidence in the activity outcomes related to physical testing of environments and processes with authentic task execution. Leaders linked this with the process of turning work as imagined into work as done, experiencing challenges and failure, testing systems under stress, finding vulnerabilities and limitations and creating relevant, agreeable solutions as a key benefit of SBT.

P5- actually stepping it out with a group of people helped visualise what the roadblocks were

Leaders appreciated the independence of the SBT simulation faculty, highlighting that an independent curiosity provides new and alternative perspectives and challenges end users to ‘think aloud’, describe decision making and deeply evaluate work systems, processes and environments. Leaders described designers and other stakeholders as immersed in the process, meaning that independent perspectives ensure a thorough and robust assessment through challenging assumptions and exploring parts of the process/system not already considered. Interestingly, leaders also emphasised value in the expertise of the independent assessment to identify gaps and make recommendations for suitable SBT scenarios and activities.

P8—I think it gives you a view through a different lens… the opportunity to think about things differently

Leaders hold accountability for organisational risk and the valued role of SBT in uncovering and analysing risks. The interview discussions reveal leader insights into the pivotal role of SBT as a diagnostic tool for risk as opposed to a complete or standalone solution. Leaders described the utility of SBT in uncovering latent safety threats (LSTs), analysing the impact of LSTs, and creating of fit-for-purpose solutions through targeted replication of real-world situations.

P2—This was a high stakes activity and failure was not an option …we needed to understand the risks … and potential disruptions

### Theme 2: Collaborative design

Unsurprisingly, leaders highlighted the importance of collaboration at all stages of activity design and delivery as a key feature of successful SBT activities. In particular, leaders identified the involvement of end users as critical to activity success. They found end users were uniquely placed to understand usual workflows and workarounds in their environment and the nuanced adaptations that are made when environmental, logistic and patient variances occur.

P9—having end users there is super helpful because they know that they understand their own work as they would do it. Whereas we, we might have a vision of how things might look but not actually function

Leaders also described benefits of using SBT as an experiential orientation tool. Leaders discussed SBT as serving dual purposes of rapidly familiarising staff with environments, systems and processes and strengthening relationships and comradery among teams.

P2—we’ve tested this, we know what to do and we know we can do it’. Things can get flustered, but ‘no its ok, we’ve got our process, we’ve got it set, we’re good’

Leaders described inclusion and collaboration as creating shared understanding and promoting resilience when apparent restrictions impact workflow. These restrictions could be building or facility design, regulatory or governance requirements or resource feasibility and availability. Leaders discussed the importance of end users in testing assumptions, refining processes and creating fit-for-purpose, sustainable and relevant solutions. Leaders also discussed SBT as an opportunity to engage transparently with stakeholders and end users, creating a shared understanding of design decisions and improving tolerance for limitations and restrictions.

P8—It just brings everyone together and helps everyone understand why it is like it is and… whether changes are viable…

### Theme 3: Reporting and recommendations

Leaders desired an approachable and itemised report with recommendations that informs decision making. Leaders referred to SBT reports and recommendations as a key part of approval processes; highlighting that workforce confidence, coupled with formal reporting and actioning of recommendations, provides assurance regarding safety, risk mitigation, and service readiness.

P9—It gives those people that are approving the go-live a sense of confidence… making decisions that a third party has gone through and done the checks

Leaders described challenges associated with the completion of SBT activities and the impact of waiting for a report to be compiled. They described a tension between the timing of activities, report availability and ‘go live’ for services and processes, indicating that short time frames between report availability and ‘go live’ reduced the ability for change, remediation and rectification if timelines lacked flexibility.

P1—… the [activity] report took a while to come back. We need to be able to access the team and have a quick turnaround on the report, sometimes in the [hospital] we need to step projects up pretty quickly

Conversely, leaders described benefits of being present during debriefing or in summary meetings with activity faculty. They reported the insights shared during these moments prepared them for the report recommendations (this was especially valuable in settings of unfavourable outcomes, failures and significant issues were encountered), enabling key stakeholders to commence actions identified in the recommendations promptly.

P5—I actually got more out of the informal huddle at the end then sometimes as a report because the narrative helped me understand a little bit more than if I had read it on a pure action plan

Leaders repeatedly discussed the applicability of the outcomes and recommendations made within SBT activities. They recognised that healthcare and health service delivery occur within a dynamic complex system yet preferred recommendations about the narrow focus or remit of the activity being conducted, with broader recommendations interpreted as a frustration due to perceived irrelevance, low priority status and a complicating factor straying from activity goals and intentions. Leaders acknowledged the tension between the desire to optimise systems and resource limitations requiring prioritisation of interventions and the accountability for risks and LSTs.

P7—So looking at really… what is a patient or staff safety issue, what is nice to have rather than everything

Referring to this tension, leaders identified the report and recommendations to be a key feature in satisfaction and perceived benefit of SBT. In particular, leaders highlighted the use of an executive summary and the presentation of issues, barriers, challenges within a risk-rated matrix. In this instance, Healthcare Failure Mode and Effect Analysis (HFMEA) tool informs decision-making and prioritisation of recommendations [21] Leaders acknowledged that confidence in the risk rating matrix is undermined if end users overstate risks, delivery of activities occurs in insufficiently prepared settings, or activity goals and intentions are unclear/misaligned.

## Discussion

This study explored hospital leaders’ views of SBT. Interviews revealed an appreciation for SBT and an understanding of its value in optimising operations, enhancing collaboration and informing decision-making.

Many of the results in this study align with Weldon et al.’s taxonomy of simulation, which includes innovation, improvement, intervention, involvement, identification, inclusion and influence [7]. Leaders involved in this study described the value of activities across these categories, in particular, innovation (introduction of novel solutions, new ways of working or new systems), improvement (enhancing existing systems or aligning with best practice), involvement (engaging with individuals and groups otherwise excluded to create shared understanding), identification (diagnostic activity to explore circumstances and events) and inclusion (key stakeholders engaged and involved).

Contrary to existing literature [21] that suggests limited understanding of simulation among healthcare executives, the interviews revealed a group of healthcare leaders who were highly engaged and demonstrated an excellent understanding of SBT’s strategic applications. This difference may be explained by the relative maturity and integration of simulation service within the organisation [25]. The simulation service described by the participants has benefited from experience, evolving to meet the complex and dynamic needs of leaders, stakeholders and the broader organisation beyond educational simulation. Stømer et al. highlight the benefits and ease of ‘reaching into’ a program with established infrastructure (even when the infrastructure arises from education/pedagogically based simulation) as supporting the integration of systems testing, governance, quality improvement and professional development/education organisational functions [25].

A key finding was the optimisation of day-to-day operations as a desirable outcome of SBT. Leaders emphasised the value of SBT in improving routine work practices, processes and environments in addition to high-stakes, low-frequency events traditionally rehearsed in educational simulation. Application of simulation strategies allows stakeholders to explore service capabilities, uncover latent safety threats and validate system preparedness that promotes safety, improves efficiency, sustainability and resilience [19]. Leaders highlighted the importance of physical testing of systems, processes and environments with frontline workers as a key moment in transitioning work as imagined to work as done. Physical simulation should include undertaking tasks in real time, navigating process, system and environmental nuances to improve the quality and perceived validity of engagement, feedback and insights shared by frontline staff [8, 19].

A notable theme was the role of SBT in regulatory and licensing requirements. Leaders described employing SBT for licensing and compliance purposes, in particular to demonstrate system safety and readiness. This application is underexplored in the literature and highlights an opportunity for simulation faculty to proactively align activities with these licensing and regulatory requirements. Doing so would enhance the value of SBT, improve engagement and support meaningful integration with broader organisation credentialling, licensing and risk management strategies.

In addition to physical testing, collaborative design and delivery through meaningful participant selection and stakeholder engagement emerged during the interviews. Although this is not a new concept for simulation enthusiasts, the results highlight an alignment between simulation faculty and leaders. Leaders value the insights of a participatory design approach whereby the participant’s knowledge of the existing systems and work processes to refine and redesign of workflows and informs sustainable, fit for purpose solutions to challenges encountered [19]. Weldon defines this collaboration as inclusion and involvement [7] enabling end users to innovate and influence improvements within the system. The interview data reflects that these SBT activities lead to contextualised risk identification and mitigation, innovation of solutions, experiential orientation and understanding implementation requirements. The application of SBT to create cohesive healthcare teams, a ready workforce, was highlighted by the leaders as both an overt objective and, at times, a hidden outcome of SBT. It is reassuring to understand that leaders’ perspectives align with existing literature, highlighting that meaningful collaboration and co-design improves system design, strengthens relationships and builds resilience of the system and its people. Simulation faculty should continue to engage stakeholders and participants to ensure activities reflect the realities of clinical practice.

Although these benefits are recognised, leaders also described tension between the complexity of healthcare systems and the desire for focused outcome reporting. Leaders expressed frustration when simulation reports extend beyond the required scope, particularly when finite resources limit the feasibility of addressing recommendations. This highlights an important ethical dilemma for leaders and simulation faculty, that is, when issues are identified and risks uncovered that are not within the scope of the activity and are not able to be addressed, leaders must decide whether to remedy the finding, defer action, escalate or reject recommendations [7].

In exploring this challenge, leaders identified the effective use of transparent feedback and reporting tools as helping to inform decision making. In particular, leaders described benefits of structured reporting that includes a standardised risk rating matrix. Desirable report characteristics included simple data presentation, use of tables, graphics and colour coding of risks, barriers, and challenges to support interpretation of risk severity and inform prioritised decision-making regarding interventions and solutions. Barlow et al. describe similar strategies, adding that structured reporting with clear signalling about risk/threat identification that enables leaders to prioritise interventions and assign accountabilities eases the burden on leaders and decision makers [16].

Reporting of outcomes and recommendations is an essential element of SBT. Leaders discussed challenges with the reporting processes related to the timing of report provision, acknowledging that preparation of reports often requires diligence to ensure participant feedback and experiences are collated and accurately represented. Leaders described pressures of time-bound effective decision making and delayed report provision. Typically, leaders are not involved in the delivery of the SBT. Although this decision is made case by case, an important element of SBT requires participants’ freedom to provide authentic critique of systems, processes and environments. This often requires the absence of leaders/key stakeholders and explicit communication about deidentification during the reporting of outcomes. In this sense, leaders in this study acknowledged appreciation for a ‘wash up’ meeting or post debriefing meeting with activity faculty as an important touch point to discuss preliminary findings and recommendations prior to report provision as an effective strategy to reduce uncertainty and impact to timelines [25]. It is important for simulation faculty to maintain transparent and responsive reporting, communication and feedback strategies with leaders and stakeholders [25].

**Table:** 

**Key points for simulation faculty**: • Align SBT with leadership priorities and regulatory/compliance frameworks where appropriate • Determine transparent communication strategies with timely touch points throughout and at the completion of SBT • Structure reports, outcomes and recommendations to improve readability—use sectioning, headings, colour coding and tables as appropriate • Ensure all stakeholders and all relevant end users are represented	

## Limitations

This study has some limitations that warrant consideration. First, the simulation service in this study is mature and has been embedded as part of the simulation service provision for more than 10 years. The simulation service has evolved and adapted to meet the needs of leaders and stakeholders. The interview data may differ for simulation services not as well enmeshed with organisation systems. Second, all researchers were members of the simulation faculty team with existing working relationships with the participants. Whilst this provided rich data collection, it may also have influenced participant responses and introduced confirmation bias. Reflexivity was maintained to balance this. Thirdly, there was a risk of selection bias as the participants were purposively selected as they had previously been a key stakeholder in a SBT project or activity. This suggests that they were supportive of and had already found value in SBT. These limitations affect the generalisability of the findings, particularly for those working in less established simulation programs. Despite this, we anticipate the key points for simulation faculty will help to improve leader engagement and facilitate integration of SBT as a quality improvement strategy.

## Conclusion

This study provides valuable insights into how healthcare leaders perceive and engage with SBT as a tool for improving safety, efficiency and readiness. There is an undeniable synergy between simulation and quality improvement that is appealing to healthcare leaders. The leaders in this study demonstrated a sophisticated understanding of SBT’s potential applications beyond educational simulation, describing its role in optimising operations, ensuring system readiness, and informing decision-making. Leaders emphasised the benefits of physical testing, collaboration and participation and timely, fit-for-purpose reporting. By understanding and responding to the perspectives of healthcare leaders, simulation faculty can promote the perceived and actual value of their work, leverage quality improvement integration and support simulation service sustainability.

Looking ahead, future research should explore the generalisability of these findings across diverse healthcare settings, particularly those with less mature simulation services. Additionally, this research uncovered value in novel applications of SBT to support licensing, credentialling and regulatory compliance. Future research could explore how to integrate this within existing frameworks and standard business as usual.

## Data Availability

No datasets were generated or analysed during the current study.

## Appendix 1

Semi-structured interview prompt.

**Table:**

**V2 Semi-structure interview (adapted following first interview)** We are interested in understanding your experience with simulation- based testing; the use of physical simulation to test our systems and processes Thinking about why you engaged the service in your project What were you hoping to achieve with the testing? **Prompt:** Establish if they had an issue/problem that they needed to understand or if it was to test the vision? Was it diagnostic or interventional? In what ways did the testing support your vision/align with your expectations • What was helpful? (**Prompt**: thinking about things in a different way, problem solving approach) • What was unhelpful? (**Prompt:** timeframe, logistics, disruption, engagement?) Let’s consider the reporting and key recommendations. How did the reporting support the project/activity? **Prompts** • Did the reporting/recommendations support the team decision making? • Was the report user friendly? • Were recommendations implemented? • Why? Why not? Barriers? **Overall impression** Our aim is to evaluate the impact of the service on key stakeholders and the organisation How engaged were the staff/team members? Did SBT have a positive impact on the service? Examples? Did SBT contribute to the success of the project? In what ways? Overall, how helpful was SBT in achieving the project outcomes/goals How could this service be improved? **Prompts** 1. Would you access the simulation service again in the future?	

## Abbreviations

SBT: Simulation-based testing
LST: Latent safety threats
